# Effects of the probiotic *Lactobacillus animalis* in murine *Mycobacterium avium* subspecies *paratuberculosis* infection

**DOI:** 10.1186/1471-2180-13-8

**Published:** 2013-01-16

**Authors:** Enusha Karunasena, Paresh C Kurkure, Russell D Lackey, Kevin Wyatt McMahon, Estevan P Kiernan, Suzanne Graham, Magdy S Alabady, David L Campos, Owatha L Tatum, Mindy M Brashears

**Affiliations:** 1 Virginia Tech, MC 0477, Washington Street, Blacksburg, VA, 24061, USA; 2 ESB Canton & Main St, MS3132, TTU, Lubbock, TX, 79409, USA; 3 TTU/HHMI Undergraduate Research Program, Lubbock, TX, 79409, USA; 4 U of Illinois Urbana-Champaign 1206, West Gregory Street, Urbana, IL, 61801, USA; 5 TTUHSC, 36014th Street, Lubbock, TX, 79409, USA

**Keywords:** Probiotics, *Mycobacterium paratuberculosis*, Direct-fed microbials, Johne’s disease, Microbiota, *Lactobacillus*, Crohn’s disease, BALB/c

## Abstract

**Background:**

MAP is a suspected zoonotic pathogen and the causative agent of Johne’s Disease in cattle and other ruminant animals. With over $1 billion dollars in loss to the dairy industry due to Johne’s Disease, efforts to eliminate or reduce MAP from cattle are of importance. The purpose of this study was to determine if daily intake of probiotics could eliminate or reduce Johne’s Disease associated symptoms and pathogenesis by MAP. Post infection, animals are often asymptomatic carriers with limited shedding of the pathogen, proving early detection to be difficult. Disease and symptoms often appear 3–4 years after infection with antibiotic treatment proving ineffective. Symptoms include chronic gastrointestinal inflammation leading to severe weight-loss from poor feed and water intake cause a wasting disease. These symptoms are similar to those found in individuals with Crohn’s Disease (CD); MAP has been implicated by not proven to be the causative agent of CD. Probiotics administered to livestock animals, including dairy and beef cattle have demonstrated improvements in cattle performance and health. Our objectives included determining the benefits of *Lactobacillus animalis* (strain name: NP-51) in MAP infected BALB/c mice by evaluating systemic and gastrointestinal response by the host and gut microbiota. Male and female animals were fed 1×10^6^ CFU/g probiotics in sterile, powdered mouse chow daily and infected with 1 × 10^7^ CFU/ml MAP and compared to controls. Animals were evaluated for 180 days to assess acute and chronic stages of disease, with sample collection from animals every 45 days. MAP concentrations from liver and intestinal tissues were examined using real time-PCR methods and the expression of key inflammatory markers were measured during MAP infection (interferon-gamma [IFN-Υ], Interleukin-1α, IL-12, IL-10, IL-6, and Tumor necrosis factor alpha [TNF-α]).

**Results:**

Our results demonstrate administration of probiotics reduces production of IFN-Υ and IL-6 while increasing TNF-α and IL-17 in chronic disease; healthful immune responses that reduce chronic inflammation associated to MAP infection.

**Conclusions:**

We observed that the immune system’s response in the presence of probiotics to MAP contributes towards host health by influencing the activity of the immune system and gut microbial populations.

## Background

*Mycobacterium avium* subspecies paratuberculosis (MAP) is a suspected zoonotic pathogen, associated with a wasting disease in ruminant animals (predominantly dairy cattle) known as Johne’s Disease (JD). This disease leads to chronic gastrointestinal tract (GIT) inflammation, preventing animals from absorbing nutrients and decreased feed intake, and accompanied with severe diarrhea. Although, infection by MAP is found to occur in utero or during weaning - through milk or fecal contamination of water and feed- JD does not appear in cattle until the age of 2–10 years [1]. It invades the host through specialized ileal tissue called Peyer’s patches and then enter macrophage. After infection, MAP survives in macrophages, within the small intestine, for years without triggering any systemic response from the immune system.

The clinical stage manifests when MAP begins to spread into lymph nodes flanking the GI tract, leading MAP to spread systemically; it is at this point that the symptoms of disease begin to appear [1-4]. Antibiotics are not effective in controlling JD once symptoms begin and the disease is ultimately fatal. The cost of JD to the cattle industry is over $1 billion dollars within the dairy industry, due to higher rates of culled cattle, poor milk production or low quality products [1,2].

### MAP is a *suspected* pathogen for crohn’s disease

Equally of significance are the symptoms of disease and pathology from MAP-associated JD which are similar to Crohn’s Disease (CD) - a chronic inflammatory bowel syndrome occurring in humans. Immunocompromised patients - such as AIDS patients - are susceptible to MAP infection [1,2,5,6]. MAP is linked (though not confirmed) to cause CD [1,7]. Many CD patients harbor MAP in their GIT tissues [8]. Introduction of subclinical animals with JD to isolated communities has demonstrated an increase in the population of JD in other livestock animals followed by increases in CD in the human population [7]. Additionally, therapies used to treat JD have been found to be effective with treatment of some CD conditions, further demonstrating associations between to the two conditions [1,7,9,10].

### MAP-induced chronic gut inflammation

Once MAP enters macrophages, the host’s immune response ‘walls-off’ the infection with the accumulation of mostly other macrophage, forming a circular-shaped granuloma- characteristic of infection [1,2,10]. MAP induces cell-mediated immune response via T-helper-1 (Th1) cells, leads to increased production of IL-1, INF-γ, IL-6, and IL-12 family cytokines which stimulate more macrophage to the site of acute-infection [1,8,11,12]. Though MAP cells are killed by macrophages, more cells enter into macrophages and multiply, new MAP are then able to further infiltrate the GI tract; these conditions create a cycle of continuous infection and inflammation, causing lesions to expand [1]. This is followed by infected macrophages entering neighboring lymph nodes and other organs through the vascular system, causing the spread of granulomatous inflammation. Penetration of infected tissue by multitudes of lymphocytes and macrophages leads to visible thickening of intestines, this prevents absorption of nutrients and causes diarrhea [1,2]. As the disease progresses, the immune response shifts from pro-inflammatory responses to increased production of TGF-β and IL-10 which suppress Th1 activity [8,11,12]. However, IL-1α is produced constitutively by macrophage at the site of infection leading to tissue scarring and damage from reactive oxygen species (ROS) [8,11,12]. As chronic inflammation persists, an increase in IL-10 and IL-2 production follows [8,11,12].

### Direct-Fed microbials reduce gut inflammation

More recently, with the use of direct-fed microbials (DFM; probiotics) in dairy cattle producers have observed decreased rates of culled cattle and animal morbidity, through wasting. The use of probiotics in the food industry is becoming an increasingly important component to developing safer and healthier foods for the public. Probiotics are organisms that are found to contribute to systemic and gut health [13-16]. Traditionally, these organisms are classified as lactic acid bacteria (LAB) that are used to ferment foods like cheese, yogurt, wine, and meat products [15]. However, their use in the medical, agricultural and scientific community is evolving [14-19]. Probiotics used in commercial foods are mostly *Lactobacillus* sp. and *Bifidobacterium* sp. [18,20-22]. The use of these organisms offers many advantages, such as bacteriocins [14,17,19,22]. Bacteriocins are peptides or proteins that have antibiotic properties [14,17,19,22]. In addition, probiotics produce other protective compounds, like hydrogen peroxide, benzoic acid, lactic acid, and biogenic amines (from the decarboxylation of amines), which decrease food-borne pathogen viability [13,18,19]. Also, tumor suppression studies in murine breast cancer models have demonstrated that fermented milk products by *Lactobacillus* sp. are able to diminish the size of tumor growth and induce increased production of antitumor immune responses [14,23,24]. These studies reveal reductions in inflammatory-mediated diseases by beneficial microbes found in food products. Studies conducted by M.M. Brashears and associates have demonstrated health benefits and improved performance by cattle fed NP-51; NP-51 has been demonstrated to reduce *Escherichia coli* O157 and *Salmonella* species shedding [16,25]. Currently, NP-51 is used by the dairy and beef industries as a direct-fed microbial. For these reasons, we decided to use NP-51 as a DFM in this study.

Our hypothesis for this study is that probiotics will contribute towards the reduction or elimination of chronic inflammation associated with symptoms of Johne’s Disease that are produced by MAP. Our objective was to evaluate animal health in an *in vivo* murine model infected with MAP and fed probiotics (*Lactobacillus animalis*; NP-51), and to further assess changes in the following: MAP concentrations in target tissues (liver and intestinal), pathology, the expression of key inflammatory markers, and changes to the gut microbiota.

## Results & discussion

### MAP concentrations in intestinal and liver tissues

**Figure 1:**
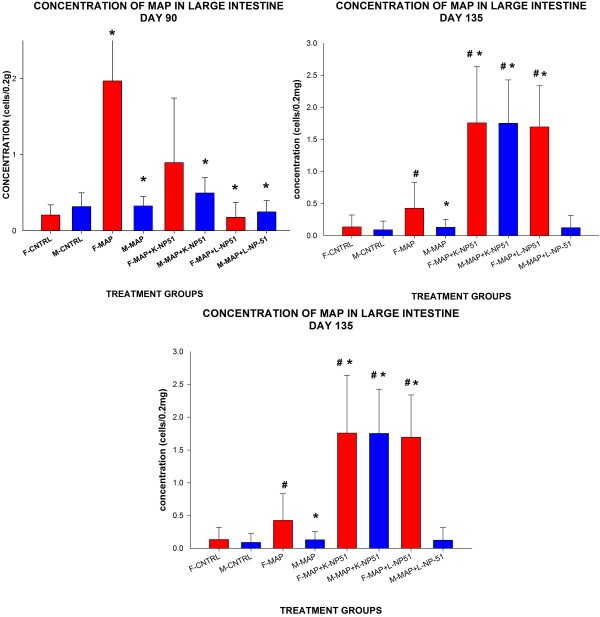
**qRT-PCR Assay to Quantitate MAP from Infected BALB/c Mouse Tissues. **Concentrations were determined using qRT-PCR analysis from large intestine and liver; The experimental groups analyzed were the following: Control (CNTRL); viable MAP (MAP); viable MAP with non-viable (killed) NP-51 (MAP + K-NP-51); viable MAP with viable (live) NP-51 (MAP + L-NP-51). For each experimental group n = 4. A: MAP Concentration in Large Intestinal Tissues. At DAY 180- there was a significant difference ‘*’ (P ≤ 0.05) between the following: males (M) with viable MAP compared to M with viable MAP and fed live NP-51 -MAP + L-NP-51. At DAY 135- several groups demonstrated significant differences including: between M MAP versus MAP + NP-51 (both L and K); similarly in females (F) in the same experimental groups were significantly different ‘*’ P ≤ 0.05; there were also notable differences ‘#’ (P ≤ 0.05) between M and F in the experimental groups MAP + NP-51 (both L and K). At DAY 90 among F, there was a significant difference ‘*’ (P ≤ 0.05) between MAP v. MAP + L-NP-51; between the sexes – F-MAP vs. M-MAP and M-MAP + L-NP-51. Animals that were infected with viable MAP (L-MAP) and viable or non-viable NP-51 (L or K NP-51) demonstrate less MAP viability at Day 90 compared to similar experimental conditions at Day 135 or Day 180; however there was no statistical difference between these differences at DAY 90. Concentrations of MAP in the large intestine were low. Additionally, there was no pathology associated with MAP infection in the intestinal tissues of animals infected with viable or non-viable MAP. These data demonstrate that there may be associations to sex in MAP infectivity of the intestinal tissues; however, to elucidate a clear correlation, further experiments will be conducted.

**Figure 2:**
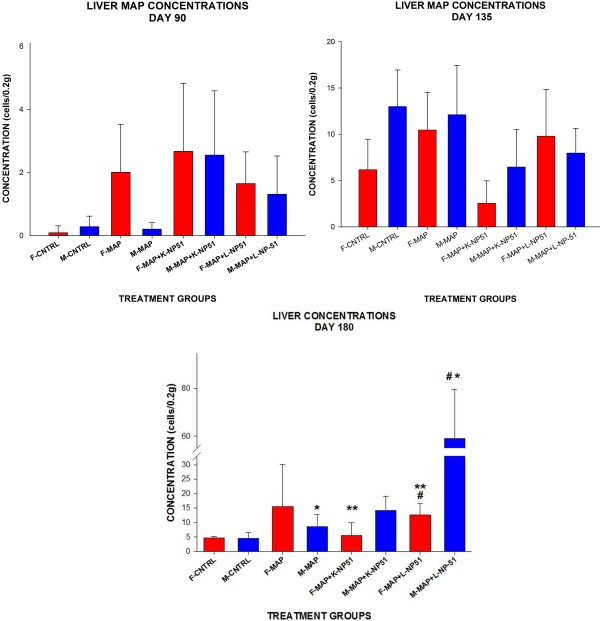
**qRT-PCR Assay to Quantitate MAP Cells from Infected BALB/c Mouse Tissues. **B: MAP Concentrations in Liver Tissues. Similar to data from large intestinal tissues, liver samples from MAP infected animals at Day 90 demonstrated the least concentration of cells from animals fed viable or non-viable NP-51. Female mice infected with viable MAP and fed viable NP-51 demonstrated less cells compared to MAP infected animals at Day 90, 135,and 180- however these results were not significantly different. Day 135 Control animals were contaminated with MAP as evidenced by histopathology (granulomas identified in liver tissues) and in these data. At DAY 180- there was a significant difference ‘*’ (P ≤ 0.05) between the following: M with viable MAP compared to M infected with viable MAP and fed live NP- 51 -MAP + L-NP-51; between M and F with MAP + L-NP-51, ‘**’ ,(P ≤ 0.05); and also, between M with MAP + L-NP-51 versus MAP + K-NP-51, ‘#’ ,(P ≤ 0.05). Histopathology analysis of liver tissues from animals infected with viable or non-viable MAP demonstrated granulomas; additionally, infected animals fed viable or non-viable NP-51 demonstrated granulomas. Similar to those data described in the large intestine, we observed differences between the sexes in MAP infectivity of the liver; also similar to those previously described- further analysis must be conducted to determine the contributive significance of this difference.

Data described in Table 1 and Figure 1 and Figure 2 reveal that MAP cells were present in intestinal tissues and the liver- organs which are associated with MAP infection and pathogenesis. Additionally, these data demonstrate that regardless of NP-51 consumption viable MAP cells were able to invade host tissues- as evidenced by granuloma formation in liver samples of animals fed viable or non-viable NP-51. However, lower concentrations of MAP cells were observed in both intestinal and liver tissues at Day 90 (45 days post MAP infection) in animals that were also fed viable or nonviable NP-51, although not significant. There were no significant changes in MAP concentrations from intestinal tissues and an increase in liver MAP concentrations were observed from Day 90 through Day 180, suggesting that MAP viability may not be deterred through the presence of probiotics (see Figure 1 and Figure 2).

**Table 1:** Total animals (n = 4) demonstrating granuloma formations in liver tissues

	**K-MAP**	**K-MAP + L-NP-51**	**L-MAP**	**L-MAP + L-NP-51**
**Day 90**	**3/4**	**3/4**	**4/4**	**4/4**
**Day 135**	2/2*	**3/4**	**4/4**	**3/4**
**Day 180**	**3/4**	**2/4**	**2/4**	**3/4**

Tissues were stained with Hemotoxylin & Eosin (H & E stain) prior to evaluation. For K-MAP samples at Day 135, only two sets of animal tissues were available for examination due to early expiration of animals before the harvest date (these data are highlighted with ‘*’). Control animals did not demonstrate granuloma formation at Day 90 and Day 180; Day 135 control animals were contaminated and were positive for granulomas in liver tissues. The data represent the number of animals that demonstrated granuloma formations per total animals examined (n =4). Experimental groups included are the following: animals fed normal chow and infected with viable MAP cells (Live-MAP; L-MAP); animals fed viable probiotics in chow and uninfected (Live NP-51; L-NP-51); animals fed viable probiotics in chow and infected with non-viable MAP cells (K-MAP + L-NP-51); animals fed viable probiotics in chow and infected with viable MAP cells (L-MAP + L-NP-51). These data demonstrate MAP infection of tissues regardless of viable or non-viable NP-51 consumption. Additionally, these data evidence that host tissues produce granulomas from exposure to K-MAP antigens.

### Host immune response to MAP

#### Cytokine production by the host to MAP versus MAP with NP-51 (probiotic)

**Figure 3:**
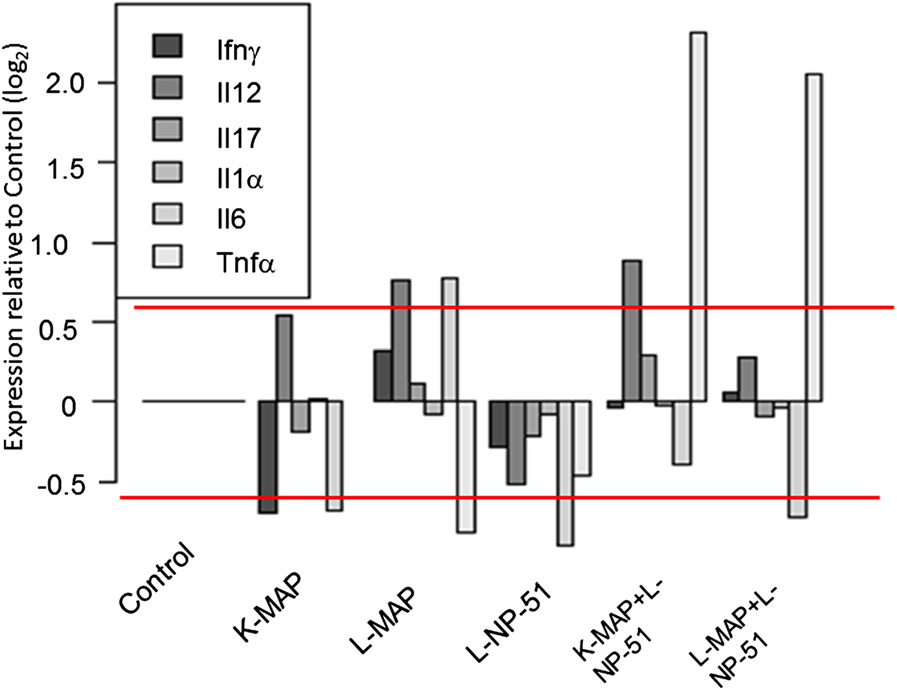
**Serum cytokine abundance relative to controls and associated with chronic MAP infection. **Data for male and female animals and time points were combined for each experimental group (n = 24) for these results. Experimental groups analyzed were the following: control animals fed normal chow and uninfected (Control; C); animals fed normal chow and infected with non-viable MAP cells, (Killed-MAP; K-MAP); animals fed normal chow and infected with viable MAP cells (Live-MAP; L-MAP); animals fed viable probiotics in chow and uninfected (Live NP-51; L-NP-51); animals fed viable probiotics in chow and infected with non- viable MAP cells (K-MAP + L-NP-51); animals fed viable probiotics in chow and infected with viable MAP cells (L-MAP + L-NP-51). Data analysis methods are further described in the data analysis section.

**Figure 4:**
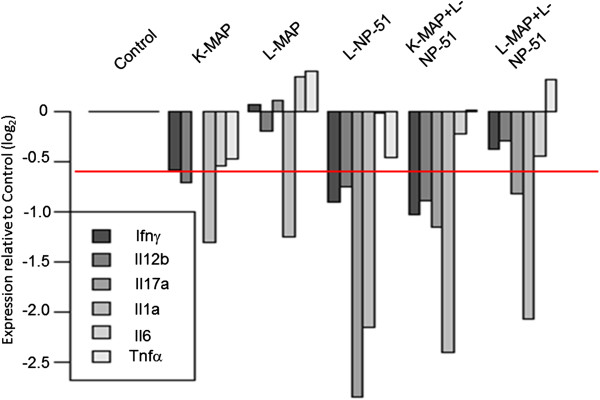
**Tissue cytokine transcript abundance relative to controls and associated with chronic MAP infection. **Data for male and female animals, time points, and tissues (small/large intestine and liver) were combined for each experimental group (n = 24). Experimental groups analyzed were the following: animal fed normal chow and uninfected (Control; C); animals fed normal chow and infected with non-viable MAP cells, (Killed-MAP; K-MAP); animals fed normal chow and infected with viable MAP cells (Live-MAP; L-MAP); animals fed viable probiotics in chow and uninfected (Live NP-51; L-NP-51); animals fed viable probiotics in chow and infected with non-viable MAP cells ( K-MAP + L-NP-51); animals fed viable probiotics in chow and infected with viable MAP cells (L-MAP + L-NP-51). Data analysis methods are further described in the data analysis section.

Cytokines measured through a multi-plex antigen capture assay (Mouse Cytokine 20-Plex Panel; Invitrogen) or transcript expression assays were focused towards inflammatory markers associated with MAP infection, including interferon-gamma (IFN-Υ), TNF-α, IL-1 α, IL-12, IL-6, and IL-17. To determine changes in cellular activity within tissues due to viable or non-viable MAP and the introduction of NP-51 we preformed assays to measure host transcript expression for key inflammatory markers. Host immune cells may produce and store non-specific, pro-inflammatory cytokines in the event of infection and yield more specific cytokines as disease progresses. For these reasons, our evaluation of cytokine transcript concentrations was to determine their active production, post MAP infection. These results are highlighted in Figures 3 and 4, respectively.

With viable MAP (L-MAP) infection, the immune response produced is characteristic of Th1 cell responses to intracellular pathogens with the production of IFN-Υ, IL-6, IL-12 (as described in Figure 3) [1,2,8]. In animals that were infected with viable MAP and fed viable probiotics (L- MAP + L-NP-51) – there is IFN-Υ production likely due to intracellular infection by MAP but this response is weaker compared to animals infected only with viable MAP, (see Figure 3). Equally, IL-12 levels are elevated but with NP-51 consumption we again observe a decrease in IL-6 circulation and an increase in pro-inflammatory cytokine- TNF-α. However, with viable-MAP infection, IL-17 circulation decreases.

IL-17 is a member of the IL-12 family; as IL-12 production increases Th17 cells are activated, producing a more selective, pathogen-associated immune response [23,24,26,27]. Our data demonstrate that animals infected with viable MAP have higher levels of IL-17 transcript expression compared to all other experimental groups (see, Figure 4). In animals infected with viable MAP and fed viable probiotics there is decreased suppression of IL-17, although IL-12 decreases. This compared to animals injected with nonviable MAP or animals fed L-NP-51 alone, further demonstrating that NP-51 is contributing towards a beneficial immune response in the host against viable MAP. Additionally, animals injected with nonviable MAP (K-MAP) and fed L-NP-51 demonstrated IL-17 expression, possibly due to increased IL-12 activity. As IL-12 circulation decreased, IL-17 also decreased. Furthermore, in the presence of NP-51 the host is able to increase TNF-α production, a pro-inflammatory response that normally decreases in chronic MAP infections to evade host immune activity. This increase in TNF- α circulation in animals fed L-NP-51 and infected with L-MAP or injected with K-MAP correlates with a decrease in IL-6 a cytokine that contributes to tissue damage in chronic inflammatory diseases, including MAP [20-23]. These results are described further in Figures 3 and 4.

Distinguishing immune responses to viable versus nonviable MAP demonstrates unique cytokine profiles for K-MAP (but absent for L-MAP). Animals injected with nonviable MAP show increased expression of IL-12 and IL-1α; however, without intracellular pathogenesis IFN-Υ and IL-6 were not present (see Figure 3). However, in animals that were injected with nonviable MAP and fed viable probiotics (K-MAP + L-NP-51), IFN-Υ remained low, likely because there is no intracellular infection. Yet, there is IL-12 production with K-MAP, possibly due to immune responses produced against circulating MAP antigens (Figure 3).

### Host immune response to probiotic (NP-51)

Similar to previous studies on probiotic strains of Lactobacilli, these data (see Figure 3) suggest that NP-51 contributes to host regulation of immune response by shifting reactions toward homeostasis by increasing or decreasing pro and anti-inflammatory pathways [16-22].

Unlike animals that received K-MAP only, those injected with K-MAP and fed L-NP-51 had increased circulation of IL-17 and TNF-α with decreased production of IL-6 (see Figure 3). In the presence of K- MAP, NP-51 increased pro-inflammatory responses (higher expression of TNF-α and IL-17) and inhibit IL-6; IL-6 causes chronic inflammatory damage during MAP infections [1,2,11]. Animals injected with K-MAP demonstrate a decrease in transcript production for all cytokines relative to controls (Figure 4). However, with L-MAP there is an increase in IFN- Υ, IL-17, IL-6, TNF- α, and decreased gene suppression of IL-12. With L-NP-51, further gene suppression for all cytokines relative to control and all other experimental groups are reported (see Figure 4). With animals injected with K-MAP and fed L-NP-51, there is decreased suppression of IL-6, TNF- α, and IL-17 compared to animals fed NP-51 alone; this may be due to the presence of K-MAP antigen inducing chronic inflammatory markers. In animals infected with L-MAP and fed NP-51 (similar to K-MAP + L-NP-51) there is decreased suppression of gene transcription for IL-17, IL-6, and TNF- α; additionally, compared to L-MAP alone, L-MAP + L-NP-51 animals have decreased IL-6 production.

It is known that concentrations of circulating cytokines and their transcript levels are not strongly correlated, suggesting that immune cells produce and store early response cytokines and chemokines, such as TNF-α, IL-1, and INF-Υ. However, as a pathogen persists the host begins to transcribe more specific cytokines, such as IL-17, IL-6, or IL-12, in addition to early response cytokines [9,24,26-29]. Our studies demonstrate that the administration of NP-51 alone down-regulates all of the studied cytokines, relative to control (Figure 4). There is an increase in TNF-α transcript expression in animals fed L-NP-51 that were also infected with L-MAP or injected with K-MAP; these results are similar to serum-cytokine results (see Figure 3 and 4). This further highlights the contributive role of NP-51 in host pro-inflammatory responses, in animals with MAP. Additionally, with animals fed L-NP-51 and infected with L-MAP there is increased repression of IL-6 transcript production compared to L-MAP infected animals- further demonstrating beneficial immune responses by NP-51 in chronic MAP associated inflammation. Comparable to serum cytokine results, transcript expression by animals fed L-NP-51 and infected or injected with L-MAP or K-MAP demonstrate a shift towards homeostasis in immune activity by producing pro and anti-inflammatory responses. These data are presented in Figures 3 and 4.

### Associations between immune response and gut microbiota

**Figure 5:**
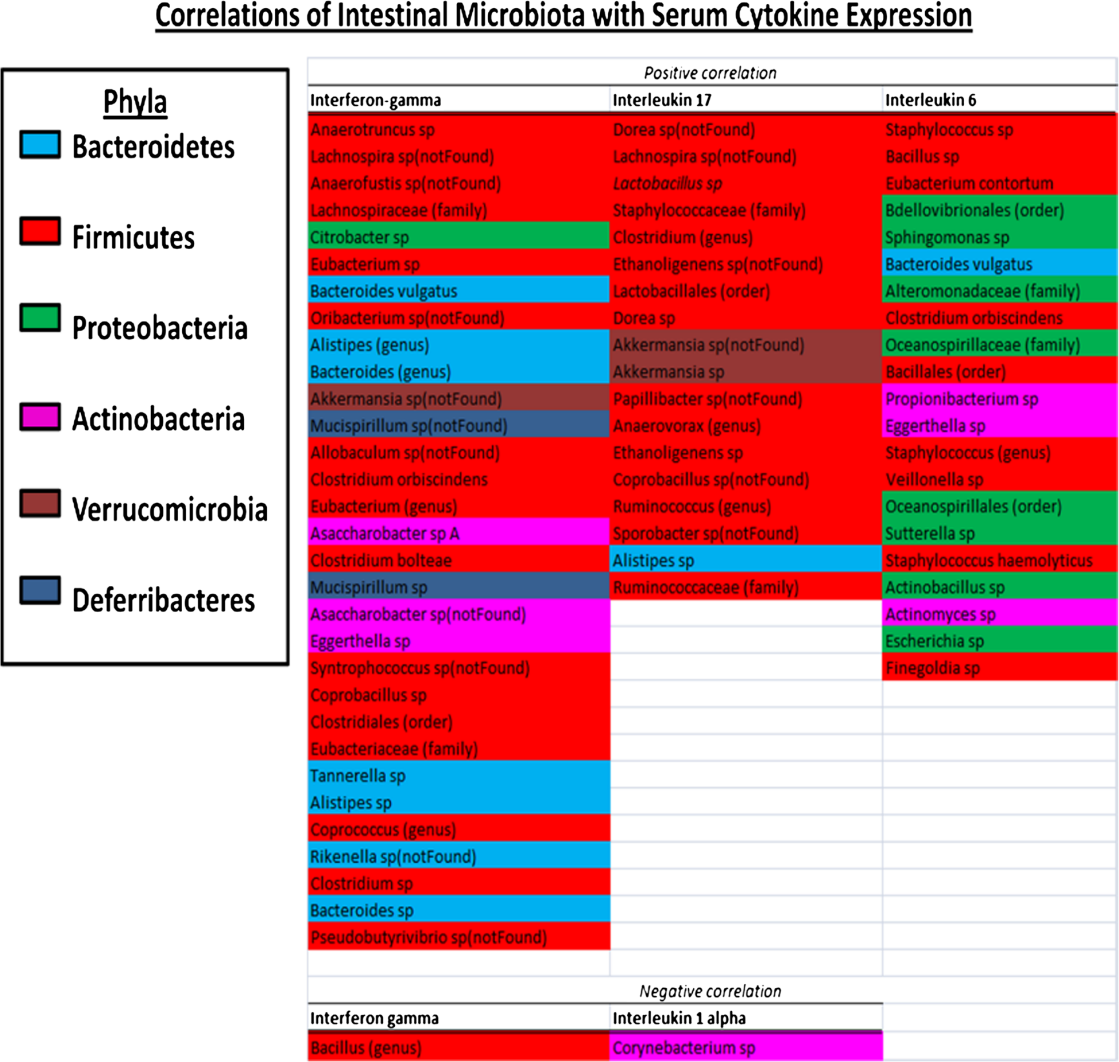
**Correlations between the relative abundance of bacteria with cytokine expression. **Bacterial family, order, genus, and species are organized into phyla- each phylum is designated by a color. *Lactobacillus* species organisms belong to the phylum *Firmicutes* (red). *Mycobacterium* species belong to the phylum *Actinobacteria* (pink). There were positive correlations with the described phyla and the presence of IL-17 and IL-6, negative correlation with IL-1α, and both positive and negative correlations with IFN-Υ. IFN-Υ, IL-1α and IL-6 are associated with MAP infections and Th-1 response [1,11]. IL-17 is associated with Th-17 cells, but is associated with IL-12 family cytokines which are produced during MAP infections [9]. Those cytokines not listed did not demonstrate any correlation with changes in the microbiota. Organisms belonging to the phylum *Bacteriodetes* were found to be mostly associated with IFN- Υ regulation. Organisms associated to *Proteobacteria* were mostly linked to IL-6. Additionally, organisms belonging to *Actinobacteria* (which include MAP) were associated with IL-6 and IFN-Υ regulation with one species also associated with IL-1α. *Lactobacillus* species and others belonging to the phylum *Firmicutes* were associated with IL-17. Similar to serum cytokine and transcript data, these data demonstrate regulation of host cytokine activity based on host-microbe interaction, both by pathogenic and beneficial microbes. Data analysis methods are further described in the data analysis section.

With chronic gut inflammatory diseases the gut microbiota - in addition to host immune responses - contributes towards disease and health [17,19,24,26-29]. Our results (described in Figure 5) demonstrate a positive correlation between gut microbiota and host immune responses, which can be either beneficial or harmful. With MAP infection, increases in INF-Υ and IL-6 can lead to tissue damage [1,2,8-12,24,26-32]. Additionally, shifts in gut flora can contribute to these immune responses [17,19,24,26-29]. Studies have demonstrated that human patients with irritable bowel syndrome (IBS) or colitis experience shifts in gut flora to higher concentrations of some species of *Bacterioidetes* which are associated with enhanced IL-12 or IFN- Υ production, or increases in *Proteobacteria* and decreases in *Firmicutes* due to increases in IL-6 [17,19,24,26-29]. Enhanced growth of *Actinobacteria* are also associated with dysbiosis of the gut in multiple diseases- including obesity, Type I diabetes, IBS, or Crohn’s disease [33]. MAP belongs to the phylum *Actinobacteria*[1]. Additionally, with individuals who have IBS amplified IL-17 production is found to promote healthy *Firmicutes*[24,26,28]. Similar to these studies, our data demonstrate greater populations of organisms belonging to the phylum *Bacterioidetes* associated with INF-Υ, and nearly all organisms associated with *Proteobacteria* correlating with IL-6 (see Figure 5). Thus, comparing the immune responses of our experimental groups with these data, we observe higher concentrations of INF-Υ and IL-6 in animals infected with viable MAP when compared to experimental groups fed NP-51 (L-MAP + L-NP-51 and K- MAP + L-NP-51)- therefore, animals with L-MAP demonstrate less beneficial flora and immune responses compared to groups fed probiotics (NP-51). Therefore, it is more likely that animals with L-MAP would support less beneficial immune responses and gut flora. *Actinobacteria* populations are also found to group with IL-6 production and some with INF-Υ production or IL- 1α down-regulation [24,26,28]. As such, with our cytokine expression data (Figure 3) we see higher concentrations of IL-6 and INF-Υ expression in experimental groups with viable MAP (L-MAP) infections, when we compared these data to our gut flora- *Actinobacteria* correlate with the expression of IL-6 and INF-Υ; a less beneficial outcome for the host.

More so, *Lactobacillus* species, and predominately organisms belonging to the phylum *Firmicutes,* are associated with IL-17 production, especially when bacterocins are produced - suggesting a healthy host-microbe association with Th17 cell response and Lactobacilli [24,27]. This suggests the production of IL-17 and reduction in IL-6 and INF-Υ expression in host tissue when NP-51 is present may reduce the proliferation of *Proteobacteria* and *Bacterioidetes* organisms that otherwise contribute to chronic gut inflammation.

Our data demonstrate NP-51 to be a beneficial gut microbe which adds to systemic host health by promoting healthful microbes in the intestinal tract that produce immune responses necessary towards homeostasis of the gut and immune system- reactions essential for reducing MAP associated disease and pathogenesis.

Probiotics have a variety of contributive effects, including the regulation of inflammation and the composition of extracellular flora in the lumen of the intestine. Through our results we were able to observe such changes in the host through the addition of NP-51 to rodent diets. However, benefits towards reducing MAP- an intracellular pathogen- were less evident in this study. These results may complement other areas of probiotic research which demonstrate reductions in MAP through the use of “unconventional bacteria”- meaning probiotics able to specifically effect intracellular pathogens [34-36]. Recent studies conducted by Click et al., show reductions in MAP concentrations in dairy cattle through the use *Detzia* subspecies (C79793-74) [37]. This organism is able to reduce MAP concentrations in *in utero* infected animals compared to most probiotics which effect extracellular loads [37]. Most studies on MAP have been focused toward eliminating mucosal inflammation and ulceration. Our studies on NP-51 support a variety of effects that appear to control this secondary inflammation. As such, this further reinforces ideas of combining probiotic organisms with differing mechanisms of action to benefit host health. Here, NP-51 is able to reduce gastrointestinal inflammation due to MAP infection; combining NP-51 with other successful probiotics that trigger reductions in pathogen proliferation could increase these benefits.

## Conclusions

There is compounding evidence to suggest that diseases due to chronic inflammation- including CD, autoimmune disorders, and asthma- share similar mechanisms of cell-mediated immune responses [9,23,30-32]. Several studies have shown that having symptoms of chronic inflammation: tissue swelling, high immune cell responsiveness, production of ROS contribute to increased oxidative stress - leading to harmful effects in host tissues [30-32]. As the incidence of inflammatory diseases (like asthma, atherosclerosis, diabetes, IBS and obesity) increase in Western nations, some groups have shown the early use of antibiotics can change the composition of microorganisms in the gut, causing increased T-cell mediated responses in airways that then cause asthma [27]. Some of these factors that increase during gut inflammation- like IL-6, IL-23 (IL-12 family cytokine), and transcription factor NF-κB - are also found to activate cancerous behavior [31,38]. Research demonstrates the accumulation of IL-6 in colon tumors causes’ growth and increase in tumor size [31]. The mechanisms by which beneficial microbes contribute to host health are a multifaceted and integrated system; these studies support our research and the importance of the host, their flora, and the impact of exchange by the two, to overall health.

These data further support studies that demonstrate the influence of probiotics in regulating chronic gut inflammation, pathogens, or the colonization of foodborne microorganisms (specifically, pathogenic *Proteobacteria* -*Salmonella*, *Campylobacter* species and *Escherichia coli)* by minimizing factors in the gut otherwise conducive to the proliferation of these and other bacteria belonging to *Bacterioidetes* or *Firmicutes*. Based on this study, we further hypothesize that contributions by beneficial microbes towards maintaining gut homeostasis may reduce symptoms associated with systemic disease that are produced by gut pathogens and these responses may have sex-specific differences.

## Methods

### Experimental design

A power test was used to determine the number of animals per experimental group. The coefficient of variance (CV %) was 5; the percent difference from the control was decided at 10; and P ≤ 0.05 with a power of 90%. The number of replicates per treatment was determined to be 5 mice/experimental group. Assistance in developing the experimental design was through personal communications with M.M. Galyean (TTU, Dept. of Animal & Food Sciences) and with reference to Martin, Meek, and Willeberg. 1987; *Veterinary Epidemiology Principals and Methods*; p. 45 Iowa State University Press. Ames, Iowa. In detail the study design is described in Table 2.

**Table 2:** Experimental design

**Experimental Groups**	**Time-Points (Number of Animals per sex M/F)**				**Key to Abbreviated Terms for Experimental Groups**	**Conditions Describing Experimental Group**
	Day 45	Day 90	Day 135	Day 180		
Control*	(5/5)	(5/5)	(5/5)	(5/5)	C = Control	No-MAP infection; No- probiotics in diet
L-MAP*	(5/5)	(5/5)	(5/5)	(5/5)	L-MAP = Viable MAP	MAP infection; No probiotics in diet
K-MAP	(5/5)	(5/5)	(5/5)	(5/5)	K-MAP = Non-viable MAP	Non-viable MAP antigen; No probiotics in diet
L-NP-51*	(5/5)	(5/5)	(5/5)	(5/5)	L-NP-51 = Viable Probiotics	No-MAP infection; probiotics in diet
L-MAP + L-NP-51*	(5/5)	(5/5)	(5/5)	(5/5)	L-MAP + L-NP-51= Viable MAP & Probiotics	MAP infection; probiotics in diet
K-MAP + L-NP-51*	(5/5)	(5/5)	(5/5)	(5/5)	K-MAP + L-NP-51 = Non- Viable MAP & Viable Probiotics	Non-viable MAP antigen; probiotics in diet
K-NP-51	(5/5)	(5/5)	(5/5)	(5/5)	K-NP-51= Non-viable Probiotics	Non-viable probiotics in diet
Maltodextrin	(5/5)	(5/5)	(5/5)	(5/5)	MD= Maltodextrin	Filler material in diet
K-MAP + K-NP-51	(5/5)	(5/5)	(5/5)	(5/5)	K-MAP + K-NP-51 = Non- viable MAP & Non-viable NP-51	Non-viable MAP antigen; non-viable probiotics in diet
L-MAP + K-NP-51	(5/5)	(5/5)	(5/5)	(5/5)	L-MAP + K-NP-51	Viable- MAP antigen; and non- viable probiotics in diet
**Total Animals Per Time Point 100**						

A description of each of the experimental groups, sexes, and treatments are described for one time point, each of the four time points in this study had the same experimental design. Select experimental groups were analyzed for metagenomic, transcriptomic and cytokine analysis based on histopathology results; the selected groups’ are highlighted with '*'.

For this study, 23–28 day old BALB/c mice equally divided between male and female, for a total of 410 animals were tested. (Charles River Laboratories, Wilmington, MA). Animals were acclimated for 2 weeks in the Texas Tech University (TTU) Animal Care and Use (ACU) facilities prior to experimentation and animal welfare, housing conditions, and euthanasia were according to protocols established through TTU-ACU (ACUC Approval Number: 07060–12). Five animals per experimental group were housed in sterilized cages with sterilized bedding. Animals were provided with sterile water and mouse chow, *ad libitum*. There were a total of 10 experimental groups and four time-points over the course of 180 days, sample collections were conducted at days 45, 90, 135, and 180. At day 0, five-male and five-female mice were euthanized and tissues were collected for histopathology and cryogenic preservation, to evaluate animals prior to experimentation. From day 0 through day 45 animals were fed a diet of: sterile powder chow, sterile powder chow combined with 1×10^6^ CFU/g NP-51, or heat-killed NP-51 at similar concentrations, daily. At day 45, 100 animals from 10 experimental groups were euthanized; animals were sedated with Isoflurane inhalation, followed with cardiac puncture and blood collection. The large (colon) and small intestinal tissues, stomach, and liver from male and female animals (n = 4) were preserved for histopathology analysis in 10% formalin solution in phosphate-buffered saline (PBS). Identical tissues collected from male/female mice (n = 6) were harvested and flash frozen in liquid nitrogen, followed with long term cryogenic preservation at −80°C. MAP concentrations were determined, from 0.2 g of harvested tissues, using qRT-PCR on large intestine and liver; liver tissues presented granulomas distinct to MAP infection based on histopathology analysis.

### MAP cultures and cell harvesting

MAP cultures were originally harvested from cattle at the USDA National Animal Disease Center (NADC), and kindly provided by Judith Stabel (Ames, Iowa). A single culture was shipped to TTU, in Middle Brooks H79 broth with Mycobactin (Allied Monitor, Fayette, MO), at refrigerated conditions. Cultures were grown and harvested according to conditions provided through Stabel et al., at the NADC [39,40]. MAP cells were rendered non-viable by boiling cultures for 20 min in a 65°C waterbath [40].

### NP-51 cultures and chow preparation

Freeze-dried NP-51 with maltodextrin (MD) was shipped directly to TTU by Culture Systems Incorporated (Mishawaka, IN). Twenty-gram packets were made with NP-51, individual packets were used once daily and any remaining material was discarded. Viable cultures of NP-51 were mixed into sterile, powdered mouse chow (7012 Teklad LM-485 Mouse/Rat Sterilizable Diet; Harlan Teklad Diets, Madison WI) using a KitchenAid® 5-Quart Tilt-Head Artisan Series Stand Mixer (Bed Bath & Beyond; Lubbock, TX) at setting 2 or 3, for 15–20 minutes in a BSL-2 safety cabinet (this insured even distribution of NP-51 in the powdered chow). Non-viable NP-51 was prepared by heating samples at 180°C in a dry oven for 20 min (Fisher Scientific Convection Gravity Oven; Fisher Sci, Houston, TX). Non-viable cultures were mixed with an identical mixer system, separately, using sterile bowls and utensils. Each chow was replaced daily with new feed according to experimental conditions. Animal cages and feed containers were handled under a BSL-2 safety cabinet. Feed containers were cleaned and sterilized weekly by autoclaving (121°C for 15 min), new feed containers were replaced along with sterilized cages and bedding every 3rd or 7th day. Utensils for preparing chow including bowls, mixing utensils, and glassware were cleaned daily and sterilized with baking at 180°C in a convection gravity oven at a minimum of 4 hours or overnight, before use.

### MAP infection and sampling schedule

On day 46, through intraperitoneal (IP) injection experimental groups were injected with 100 μl of sterile PBS containing 1×10^7^ CFU/ml viable or non-viable MAP. Controls were injected with 100 μl PBS only. Animals were observed closely for 48 h for negative physiological reactions to IP injections. Every 45 days post infection - Days 90, 135, and 180- necropsies were performed.

### Serum/ tissue collection & cytokine analysis

At each necropsy, blood was collected into serum separation tubes, and serum was pooled from each experimental group (n =5) (13×100 mm, SST™ Serum Separation Tubes; Beckton- Dickinson; San Jose, CA). Blood samples were refrigerated for 24-48 h after collection, followed by centrifugation at 5,000 × g for 5 min (Marathon 2100R, Thermo-Fisher Scientific; Houston, TX). Serum was transferred, using disposable, sterile serological pipettes to sterile, 2 ml cryogenic tubes and stored at −20°C (Fisher Scientific; Houston, TX).

Two-hundred microliters of serum from each experimental condition and for all collection time points were shipped to TTUHSC, at El Paso and analyzed using a Mouse Cytokine 20-Plex Panel for the Luminex® platform - according to manufacturer protocol (Invitrogen/Life Technologies; Carlsbad, CA). Serum was analyzed in triplicate wells and compared to standards.

### Tissue RNA/DNA extractions, cDNA synthesis, & cDNA analysis

Colon tissues were ground with mortar and pestle in liquid nitrogen to preserve RNA/DNA and prevent nuclease activity in tissues. Approximately, 100 mg of tissue were extracted for RNA using a Trizol® kit (Invitrogen, Carlsbad, CA). Co-purification of DNA from these extractions were preformed from the separated organic layer, using a DNeasy® Blood & Tissue Kit according to protocols for total bacterial DNA extractions (Qiagen, Valencia, CA). Purified DNA were kept in 1x Tris-EDTA Buffer and concentrations were measured spectrophotometerically at a ratio of 260/280 nm (Nanodrop 1000, Wilmington, DE). DNA at concentrations of 40–50 ng/μl in 50 μl of water was provided for sequencing. High throughput sequencing was conducted using 454 ®pyrosequencing technology (Roche Laboratories, Branford, CT) at Research and Testing Laboratories, LLC (Lubbock, TX).

Duplicate samples of RNA, collected from triplicate animals from each sex for each experimental condition were prepared for quantitative Real Time- PCR (qRT-PCR). High- Capacity® cDNA Reverse Transcription kit was used (ABI, Foster City, CA). For RNA samples with concentrations below 60 ng/μl a High® Capacity RNA-to-cDNA Master Mix kit was used for cDNA synthesis (ABI; Foster City, CA). cDNA were analyzed using SYBR green probes for genes of interest for Open® Array platform (Life Technologies Inc.; Carlsbad, CA). Probes for all genes were selected from array panels and customized for our study- 9 plates were used in the analysis. Assays were performed by The University of Texas, Southwestern at Dallas. Analysis of data was conducted using Open® Array Real Time qPCR Analysis Software Version 1.0.4. Each cDNA sample was analyzed in duplicate, from triplicate animals and both sexes.

### qRT-PCR analysis of MAP concentrations from tissues

The template DNA used for construction of standards was extracted from MAP culture. Briefly, 10 ml of the MAP culture was pelleted using centrifugation (Marathon 2100R, Thermo-Fisher Scientific, Houston, TX) at 5000 × g for 15 minutes. The cells were washed twice with HPLC-grade water (Ricca Chemical Company; Arlington, TX) and again suspended in new HPLC-grade water. DNA was extracted by heating 50 μl of cell suspension in PCR tubes (VWR Int, Westchester PA) at 99°C for 15 minutes in Gene Amp PCR system 2700 Thermocycler (Applied Biosystems, Foster City, CA). The heated sample was centrifuged to pellet the cell debris and the supernatant was used as template for successive experiments.

The primers used for this assay amplifies a 163 bp region of the IS-Mav region in the MAP genome. Various primer pairs were tested before selecting the ISMav2 primers [3,4,41-43]. By using plasmids with the 163 bp fragment DNA insertion as standards, serial dilutions were tested to develop a standard curve and then enumerate the number of MAP cells in the experimental samples by plotting the Ct values on the curve. This was confirmed using the melting curve analysis of the PCR product which showed only one peak for ISMav2; thus the amplicon was very specific for MAP. Using recombinant plasmids at specific concentrations as standards we tried to do an absolute quantification of MAP but based on the standard curve generated using these plasmids the minimum number of cells that can be detected was 10. PCR reactions were carried out in 50 μl containing primer ISMav2 (Forward seq 5^′^-CGG CAA AAT CGA GCA GTT TC-3^′^; Reverse seq 5^′^-TGA GCC GGT GTG ATC TTT-3^′^), 10 μl of template DNA, using Qiagen Hot®-Start PCR kit (Qiagen Sciences, MD) following manufacturer protocols [3]. The PCR products were run on 2% agarose gel stained with EtBr in 1X TAE buffer to check for a single amplicon. The PCR product was purified using Qiagen® PCR-Purification Kit (Qiagen Sciences, MD) and used for direct cloning using pGEM-T® Easy vector system (Promega Corporation, Madison, WI) in HB101® competent *E. coli* cells (Promega Corporation, Madison, WI) following manufacturer’s protocol. The recombinant plasmids were purified using Quick ®Plasmid mini- prep kit (Invitrogen, Carlsbad, CA) following manufacturer’s methods and were sequenced at the Biotech Core Facility (Texas Tech University, Lubbock, TX). The sequence data was analyzed using BLAST to confirm its uniqueness to MAP. These recombinant plasmids were used as standards for RT-PCR.

The plasmid concentration was measured at 260 nm at a ratio of 260/280 nm using ND®-1000 spectrophotometer in the TTU Biotech Core Facility. Based on the concentration and the length of the recombinant plasmids, the number of plasmids in the solution was calculated and dilutions of 10, 100, 1000, and 10000 plasmids per microliter were prepared in 1X TE buffer.

These plasmid dilutions were used for constructing a standard curve for the quantification of MAP cells from mouse colon and liver tissue using RT- PCR. A 16 s rRNA sequence present in bacteria was used as the reference gene. The primer pair used for amplification of that sequence were universal primers (Forward 5^′^ CCA TGA AGT CGG AAT CGC TAG-3^′^; Reverse 5^′^- ACT CCC ATG GTG TGA CGG-3^′^).

PCR reactions were carried out in 25 μl using SuperScript® III Platinum Two step qRT- PCR kit with SYBR Green (Invitrogen; Carlsbad, CA). The reaction set up and the thermal cycling parameters were according to manufacturer’s instructions. The 7500 Real-Time PCR system (Applied Biosystems; Foster City, CA) at the TTU, Biotech Core Facility was used for real time detection of amplified dsDNA with SYBR Green. Melting curve analysis was also performed according to the instrument protocol. The experimental samples were divided into 4, 96 well plates. Every sample was run in triplicate. Each plate had non-template controls for ISMav2 primers and universal primers; quantification standards were recombinant plasmids with ISMav2 representative of cell numbers (1×10^5^, 1×10^3^, 1×10^2^, and 1×10^1^), experimental samples were evaluated with ISMav2 primers or universal primers. Specific amplification of target DNA was monitored by comparing the normalized reporter signal (SYBR Green) for a threshold cycle (Ct) and the signal obtained for controls.

### Data analysis

#### Real time PCR analysis

Data were normalized and a One Way Analysis of Variance (ANOVA) was conducted; if the mean values among the treatment groups were greater than what would be expected by chance there was a significant difference (P < 0.05). A post-hoc, all pairwise multiple comparison procedure (Tukey Test) was performed for statistical analysis of significance.

#### Tissue cytokine transcript analysis

Files from the Luminex® and Open® Array analyses were parsed and organized into tab-delimited files using custom perl scripts. Values across multiple days and sexes were averaged to result in one value for each of 6 experimental conditions (Control, L-MAP, K-MAP, L-NP-51, K-MAP + L-NP-51 and L-MAP + L-NP-51). Targets (cytokines or transcripts) that gave reliable results above background were included in the final analysis. All values were normalized to control values and expressed as log base 2.

#### Gut microbiota analysis

For microbiota analysis, .sff files generated from 454 sequencing were demultiplexed, converted to .fastq files and resulting sequences were trimmed and mapped to 16S ribosomal DNA intergenic regions to classify the origin of the sequence. The methodology associated with 454 sequencing were conducted by Research and Laboratory Testing (Lubbock, TX) according to protocols previously developed and described by Dowd et al., [44]. Sequencing data were deposited to GenBank short reads archive (SRA056455). The percent of sequences from each organism in each sample was normalized across all samples and final values were normalized to control and values were expressed as log base 2 of the difference between each sample and the control. A custom R script was written to perform a Pearson correlation between the relative abundance of each genus and relative abundance of each cytokine; geni with p-values of <0.05 in the Pearson and at least one cytokine from the Luminex® analysis were included in the final table, separated based on whether the r-value was positive (positive correlation) or negative (negative correlation).

## Abbreviations

MAP: Mycobacterium avium subspecies paratuberculosis L-MAP- Live MAP (viable cells); K-MAP: Killed MAP (non-viable cells) L-NP51- live NP51 (viable NP51 cells) K-NP51- killed NP51 (non-viable cells); F: Female; M: Male.

## Competing interests

The authors declare that they have no competing interests.

## Authors’ contributions

EK: Designed, coordinated and conducted the animal studies, histopathology, molecular/microbiology and immunology assays, analysis and interpretation of data/results and drafted the manuscript. PCK: Tissue collections, DNA/RNA extractions from tissues, qRT-PCR assays to quantitate MAP from intestinal tissues, and drafted a section of the manuscript on RT-PCR analysis of MAP. RDL: Conducted animals feeding regimen, tissue collections, DNA/RNA extractions from tissues. KWM: Contributed to the design of qRT-PCR assays, tissue collection procedures, RNA/DNA extractions, and conducted the analyses of data for immune and microbiota assays; additionally, he drafted a section on methods for data analysis. EPK: Conducted animals feeding regimen, tissue collections, and immune cell analysis through Giemsa staining. SG: Conducted and interpreted histopathology for all animals tissues examined. MSA: Conducted the analysis of microbiota data collected through high-through put next generation sequencing methods. DC: Conducted qRT-PCR assays on liver tissues to quantitate MAP OLT: Contributed in the coordination and conduction of PCR, qRT-PCR assays on MAP. MMB: Contributed in the design and coordination of NP-51/probiotic use in the animal model, methods for probiotics intake, microbiology analysis of probiotics/MAP. All authors read and approved the final manuscript.
